# Eagle syndrome

**DOI:** 10.4103/0971-3026.50826

**Published:** 2009-05

**Authors:** Deepika Raina, Rajesh Gothi, Sriram Rajan

**Affiliations:** Department of Radiodiagnosis and Imaging, Diwan Chand Satyapal Aggarwal Imaging Center, New Delhi, India

**Keywords:** Ossification, stylohyoid ligament, styloid process

## Abstract

Eagle syndrome occurs due to elongation of the styloid process or calcification of the stylohyoid ligament, which then may produce a pain sensation due the pressure exerted on various structures in the head and neck. When suspected, imaging helps in identifying the abnormally elongated styloid process or the calcified ligament. In recent years, three-dimensional CT (3DCT) has proved to be valuable in these cases. We report the case of a 62-year-old man with this syndrome in whom imaging with 3DCT conclusively established the diagnosis.

Eagle syndrome is a condition in which there is a painful sensation in the head and neck region due to elongation of the styloid process or calcification of the stylohyoid ligament. It is a rare condition and most of the patients may be asymptomatic; however, when these ossified structures exert pressure on the various structures in the head and neck there can be a wide range of symptoms, including pharyngeal discomfort, painful neck movements, change in voice, painful tongue movements, increased secretion of saliva, otalgia, and headache.[1]

Radiographs of the skull, both anteroposterior and lateral views, can reveal the elongated styloid process, but the superimposition of various other structures often makes diagnosis difficult. The development of three-dimensional CT (3DCT) has made possible better depiction of the anatomy of the surrounding structures. 3DCT is of great help when surgical correction is being contemplated.[2]

## Case Report

**Figure 1 (A, B):**
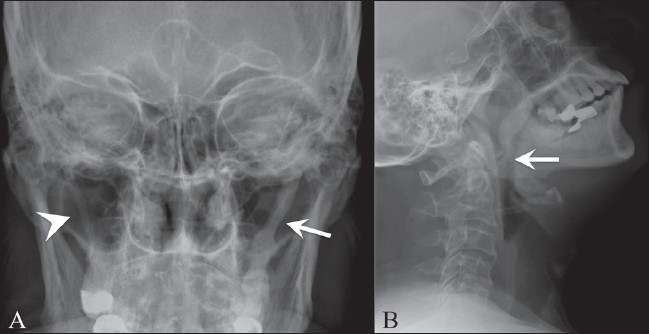
Antero-posterior (A) and lateral (B) radiographs of the skull show elongated styloid processes on both sides; there is a completely calcified stylohyoid ligament on the left side (arrow) and a partially calcified one on the right side (arrowhead)

A 62-year-old male presented with 6 months’ history of pain in the throat, radiating to the head and neck; the pain was more on the left side, especially on turning the head towards the left. Anterior and posterior radiographs of the skull showed elongated, excessively ossified styloid processes on both sides, with the left being more affected than the right [Figure 1]. There was also calcification of the stylohyoid ligaments on both sides, the right being partially calcified and the left completely calcified.

**Figure 2 (A, B):**
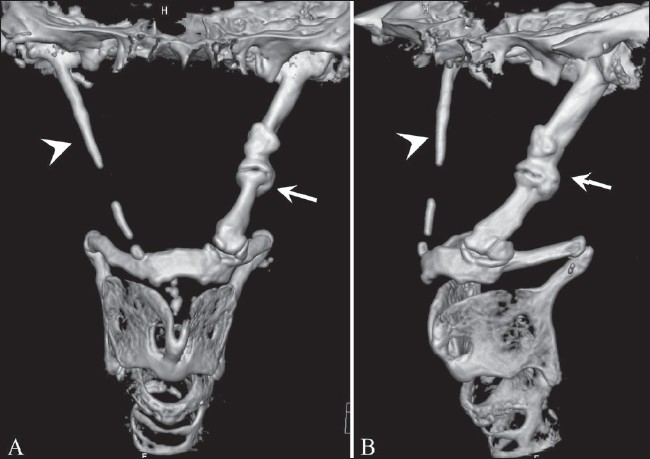
Antero-posterior (A) and oblique (B) 3DCT images show the stylohyoid chain apparatus, with elongated styloid processes on both sides; there is a completely calcified stylohyoid ligament on the left side (arrow) and a partially calcified one on the right side (arrowhead)

The patient also underwent a CT scan (Sensation 64, Siemens, Erlangen, Germany) of the head and neck; images were obtained in the axial, coronal, and sagittal planes and three-dimensional (3D) reconstructions were done. The CT scan showed excessive ossification of both styloid processes leading to an increase in their length and thickness; the left styloid was more affected than the right. There was calcification of both stylohyoid ligaments, which was partial on the right and complete on the left side [Figure 2].

## Discussion

Eagle syndrome (ES) is a clinical condition in which there is abnormal ossification of the stylohyoid apparatus, consisting of the styloid process, the attached stylohyoid ligament, and the lesser cornu of the hyoid bone. Anatomically, the styloid process arises from the temporal bone and passes downwards, forwards, and medially. Embryologically, it is derived from the Reichert's cartilaginous component of the second branchial arch. Ossification of the styloid process and the stylohyoid ligament leads to an increase in the thickness and length of the styloid process, which then presses on the adjacent structures like the internal jugular vein, carotid artery, facial nerve, vagal nerve, glossopharyngeal nerve, and hypoglossal nerve, resulting in various pressure symptoms. The styloid process normally measures 2.5–3 cm in length; when the length exceeds 3 cm, it is said to be elongated.

The cause of this elongation of the styloid process is not well understood. It can be idiopathic, congenital (due to the persistence of cartilaginous elements of precursors of the styloid process), or acquired (due to the proliferation of osseous tissue at the insertion of the stylohyoid ligament).

The symptoms that patients complain of have varied pathophysiological explanations; for example, symptoms may be due to: 1) fracture of the styloid process leading to granulation tissue proliferation, which results in pressure on surrounding structures; 2) compression of adjacent nerves, eg, the glossopharyngeal, the lower branch of the trigeminal, or the chorda tympani; 3) degenerative and inflammatory changes at the tendinous portion of the stylohyoid insertion, which is known as insertion tendonitis; 4) irritation of the pharyngeal mucosa due to direct compression, or post-tonsillectomy scarring involving the cranial nerves V, VII, IX, and X; and 5) impingement of the carotid vessels with irritation of the sympathetic nerves in the arterial sheath.

There are two types of ES as described originally by Eagle: first is the classic styloid process syndrome due to fibrous tissue causing distortion of the cranial nerve endings in the tonsillar bed after tonsil removal; and a second type due to compression of the sympathetic chain in the carotid sheath.[3]

ES can be diagnosed radiologically and by physical examination. The elongated styloid process can be felt in the tonsillar fossa, and palpation can lead to an increase in symptoms. This elongation can be confirmed radiologically using conventional radiographs or CT scan.[4] 3DCT helps in surgical planning and allows the physician to better explain the lesion and the surgical details to patient. It also helps to obtain measurements in three dimensions, along the plane of the styloid process being measured; in contrast there is underestimation of the length of the styloid process with two-dimensional cross-sectional imaging, where even in the coronal plane, the images are usually not parallel to the styloid process.

ES can be treated by surgical and nonsurgical means. Nonsurgical treatments involve reassurance to the patient, analgesics, and steroid injections. Surgical treatment can be performed using one of two approaches: transpharyngeal or extraoral. The latter is thought to be superior because it is less likely to cause deep space infection of the neck.[4]
